# Supplementary material for: Interaction of insecticidal proteins from Pseudomonas spp. and Bacillus thuringiensis for boll weevil management
Source: PLoS One. 2023 Nov 30;18(11):e0294654. doi: 10.1371/journal.pone.0294654 (PMC10688866; doi:10.1371/journal.pone.0294654)
Supplement: S1 File — (DOCX) [file pone.0294654.s001.docx]

| **Bioassay *A. grandis* - IPD072Aa Proteins** | | | | | | |
| --- | --- | --- | --- | --- | --- | --- |
| IPD072Aa | | | | | | |
| treatment | repetition | total | alive | dead | Mort (%) | (X/100)^0.5 |
| Cont | 1 | 16 | 16 | 0 | 0 | 0,00 |
| Cont | 2 | 16 | 15 | 1 | 6,25 | 0,25 |
| Cont | 3 | 16 | 15 | 1 | 6,25 | 0,25 |
| 4 | 1 | 16 | 12 | 2 | 12,5 | 0,35 |
| 4 | 2 | 16 | 15 | 1 | 6,25 | 0,25 |
| 4 | 3 | 16 | 13 | 3 | 18,75 | 0,43 |
| 8 | 1 | 16 | 13 | 3 | 18,75 | 0,43 |
| 8 | 2 | 16 | 11 | 5 | 31,25 | 0,56 |
| 8 | 3 | 16 | 9 | 7 | 43,75 | 0,66 |
| 16 | 1 | 16 | 7 | 9 | 56,25 | 0,75 |
| 16 | 2 | 16 | 5 | 11 | 68,75 | 0,83 |
| 16 | 3 | 16 | 6 | 10 | 62,5 | 0,79 |
| 32 | 1 | 16 | 2 | 14 | 87,5 | 0,94 |
| 32 | 2 | 16 | 2 | 14 | 87,5 | 0,94 |
| 32 | 3 | 16 | 3 | 13 | 81,25 | 0,90 |
| 64 | 1 | 16 | 3 | 13 | 81,25 | 0,90 |
| 64 | 2 | 16 | 2 | 14 | 87,5 | 0,94 |
| 64 | 3 | 16 | 1 | 15 | 93,75 | 0,97 |
| 128 | 1 | 16 | 0 | 16 | 100 | 1,00 |
| 128 | 2 | 16 | 0 | 16 | 100 | 1,00 |
| 128 | 3 | 16 | 2 | 14 | 87,5 | 0,94 |
| 256 | 1 | 16 | 0 | 16 | 100 | 1,00 |
| 256 | 2 | 16 | 1 | 15 | 93,75 | 0,97 |
| 256 | 3 | 16 | 1 | 15 | 93,75 | 0,97 |

| **Bioassay *A. grandis* - PIP-47Aa Proteins** | | | | | | |
| --- | --- | --- | --- | --- | --- | --- |
| PIP-47Aa | | | | | | |
| treatment | repetition | total | alive | dead | Mort (%) | (X/100)^0.5 |
| Cont | 1 | 16 | 16 | 0 | 0 | 0,00 |
| Cont | 2 | 16 | 15 | 1 | 6,25 | 0,25 |
| Cont | 3 | 16 | 15 | 1 | 6,25 | 0,25 |
| 4 | 1 | 16 | 13 | 3 | 18,75 | 0,43 |
| 4 | 2 | 16 | 14 | 2 | 12,5 | 0,35 |
| 4 | 3 | 16 | 13 | 3 | 18,75 | 0,43 |
| 8 | 1 | 16 | 13 | 3 | 18,75 | 0,43 |
| 8 | 2 | 16 | 10 | 6 | 37,5 | 0,61 |
| 8 | 3 | 16 | 12 | 4 | 25 | 0,50 |
| 16 | 1 | 16 | 11 | 9 | 56,25 | 0,75 |
| 16 | 2 | 16 | 10 | 6 | 37,5 | 0,61 |
| 16 | 3 | 16 | 6 | 10 | 62,5 | 0,79 |
| 32 | 1 | 16 | 4 | 12 | 75 | 0,87 |
| 32 | 2 | 16 | 9 | 7 | 43,75 | 0,66 |
| 32 | 3 | 16 | 5 | 11 | 68,75 | 0,83 |
| 64 | 1 | 16 | 1 | 15 | 93,75 | 0,97 |
| 64 | 2 | 16 | 3 | 13 | 81,25 | 0,90 |
| 64 | 3 | 16 | 2 | 14 | 87,5 | 0,94 |
| 128 | 1 | 16 | 0 | 16 | 100 | 1,00 |
| 128 | 2 | 16 | 1 | 15 | 93,75 | 0,97 |
| 128 | 3 | 16 | 1 | 15 | 93,75 | 0,97 |
| 256 | 1 | 16 | 0 | 16 | 100 | 1,00 |
| 256 | 2 | 16 | 0 | 16 | 100 | 1,00 |
| 256 | 3 | 16 | 1 | 15 | 93,75 | 0,97 |

| **Bioassay *A. grandis* - IPD072Aa + PIP-47Aa Proteins** | | | | | | |
| --- | --- | --- | --- | --- | --- | --- |
| IPD072Aa + PIP-47Aa | | | | | | |
| treatment | repetition | total | alive | dead | Mort (%) | (X/100)^0.5 |
| Cont | 1 | 16 | 16 | 0 | 0 | 0,00 |
| Cont | 2 | 16 | 15 | 1 | 6,25 | 0,25 |
| Cont | 3 | 16 | 15 | 1 | 6,25 | 0,25 |
| 2 | 1 | 16 | 12 | 4 | 25 | 0,50 |
| 2 | 2 | 16 | 10 | 6 | 37,5 | 0,61 |
| 2 | 3 | 16 | 10 | 6 | 37,5 | 0,61 |
| 4 | 1 | 16 | 5 | 11 | 68,75 | 0,83 |
| 4 | 2 | 16 | 11 | 5 | 31,25 | 0,56 |
| 4 | 3 | 16 | 7 | 9 | 56,25 | 0,75 |
| 8 | 1 | 16 | 1 | 15 | 93,75 | 0,97 |
| 8 | 2 | 16 | 5 | 11 | 68,75 | 0,83 |
| 8 | 3 | 16 | 3 | 13 | 81,25 | 0,90 |
| 16 | 1 | 16 | 2 | 14 | 87,5 | 0,94 |
| 16 | 2 | 16 | 0 | 16 | 100 | 1,00 |
| 16 | 3 | 16 | 1 | 15 | 93,75 | 0,97 |
| 32 | 1 | 16 | 1 | 15 | 93,75 | 0,97 |
| 32 | 2 | 16 | 0 | 16 | 100 | 1,00 |
| 32 | 3 | 16 | 1 | 15 | 93,75 | 0,97 |
| 64 | 1 | 16 | 0 | 16 | 100 | 1,00 |
| 64 | 2 | 16 | 1 | 15 | 93,75 | 0,97 |
| 64 | 3 | 16 | 0 | 16 | 100 | 1,00 |
| 128 | 1 | 16 | 0 | 16 | 100 | 1,00 |
| 128 | 2 | 16 | 0 | 16 | 100 | 1,00 |
| 128 | 3 | 16 | 1 | 15 | 93,75 | 0,97 |

| **Bioassay *A. grandis* - Cry1Ia10 Proteins** | | | | | | |
| --- | --- | --- | --- | --- | --- | --- |
| Cry1Ia10 | | | | | | |
| treatment | repetition | total | alive | dead | Mort (%) | (X/100)^0.5 |
| Cont | 1 | 16 | 16 | 0 | 0 | 0,00 |
| Cont | 2 | 16 | 16 | 0 | 0 | 0,00 |
| Cont | 3 | 16 | 15 | 1 | 6,25 | 0,25 |
| 4 | 1 | 16 | 13 | 3 | 18,75 | 0,43 |
| 4 | 2 | 16 | 12 | 4 | 25 | 0,50 |
| 4 | 3 | 16 | 13 | 3 | 18,75 | 0,43 |
| 8 | 1 | 16 | 11 | 5 | 31,25 | 0,56 |
| 8 | 2 | 16 | 12 | 4 | 25 | 0,50 |
| 8 | 3 | 16 | 7 | 9 | 56,25 | 0,75 |
| 16 | 1 | 16 | 5 | 11 | 68,75 | 0,83 |
| 16 | 2 | 16 | 6 | 10 | 62,5 | 0,79 |
| 16 | 3 | 16 | 1 | 15 | 93,75 | 0,97 |
| 32 | 1 | 16 | 3 | 13 | 81,25 | 0,90 |
| 32 | 2 | 16 | 1 | 15 | 93,75 | 0,97 |
| 32 | 3 | 16 | 2 | 14 | 87,5 | 0,94 |
| 64 | 1 | 16 | 1 | 15 | 93,75 | 0,97 |
| 64 | 2 | 16 | 2 | 14 | 87,5 | 0,94 |
| 64 | 3 | 16 | 1 | 15 | 93,75 | 0,97 |
| 128 | 1 | 16 | 1 | 15 | 93,75 | 0,97 |
| 128 | 2 | 16 | 1 | 15 | 93,75 | 0,97 |
| 128 | 3 | 16 | 0 | 16 | 100 | 1,00 |
| 256 | 1 | 16 | 0 | 16 | 100 | 1,00 |
| 256 | 2 | 16 | 0 | 16 | 100 | 1,00 |
| 256 | 3 | 16 | 1 | 15 | 93,75 | 0,97 |

| **Bioassay *A. grandis* - Cry3Aa Proteins** | | | | | | |
| --- | --- | --- | --- | --- | --- | --- |
| Cry3Aa | | | | | | |
| treatment | repetition | total | alive | dead | Mort (%) | (X/100)^0.5 |
| Cont | 1 | 16 | 16 | 0 | 0 | 0,00 |
| Cont | 2 | 16 | 16 | 0 | 0 | 0,00 |
| Cont | 3 | 16 | 15 | 1 | 6,25 | 0,25 |
| 4 | 1 | 16 | 12 | 4 | 25 | 0,50 |
| 4 | 2 | 16 | 10 | 6 | 37,5 | 0,61 |
| 4 | 3 | 16 | 14 | 2 | 12,5 | 0,35 |
| 8 | 1 | 16 | 9 | 7 | 43,75 | 0,66 |
| 8 | 2 | 16 | 5 | 11 | 68,75 | 0,83 |
| 8 | 3 | 16 | 11 | 9 | 56,25 | 0,75 |
| 16 | 1 | 16 | 6 | 10 | 62,5 | 0,79 |
| 16 | 2 | 16 | 3 | 13 | 81,25 | 0,90 |
| 16 | 3 | 16 | 3 | 13 | 81,25 | 0,90 |
| 32 | 1 | 16 | 3 | 13 | 81,25 | 0,90 |
| 32 | 2 | 16 | 2 | 14 | 87,5 | 0,94 |
| 32 | 3 | 16 | 1 | 15 | 93,75 | 0,97 |
| 64 | 1 | 16 | 4 | 12 | 75 | 0,87 |
| 64 | 2 | 16 | 2 | 14 | 87,5 | 0,94 |
| 64 | 3 | 16 | 0 | 16 | 100 | 1,00 |
| 128 | 1 | 16 | 0 | 16 | 100 | 1,00 |
| 128 | 2 | 16 | 0 | 16 | 100 | 1,00 |
| 128 | 3 | 16 | 1 | 15 | 93,75 | 0,97 |
| 256 | 1 | 16 | 0 | 16 | 100 | 1,00 |
| 256 | 2 | 16 | 1 | 15 | 93,75 | 0,97 |
| 256 | 3 | 16 | 0 | 16 | 100 | 1,00 |

| **Bioassay *A. grandis* - Cry8B Proteins** | | | | | | |
| --- | --- | --- | --- | --- | --- | --- |
| Cry8B | | | | | | |
| treatment | repetition | total | alive | dead | Mort (%) | (X/100)^0.5 |
| Cont | 1 | 16 | 16 | 0 | 0 | 0,00 |
| Cont | 2 | 16 | 16 | 0 | 0 | 0,00 |
| Cont | 3 | 16 | 15 | 1 | 6,25 | 0,25 |
| 4 | 1 | 16 | 10 | 6 | 37,5 | 0,61 |
| 4 | 2 | 16 | 10 | 6 | 37,5 | 0,61 |
| 4 | 3 | 16 | 11 | 5 | 31,25 | 0,56 |
| 8 | 1 | 16 | 3 | 13 | 81,25 | 0,90 |
| 8 | 2 | 16 | 9 | 7 | 43,75 | 0,66 |
| 8 | 3 | 16 | 7 | 9 | 56,25 | 0,75 |
| 16 | 1 | 16 | 3 | 13 | 81,25 | 0,90 |
| 16 | 2 | 16 | 2 | 14 | 87,5 | 0,94 |
| 16 | 3 | 16 | 6 | 10 | 62,5 | 0,79 |
| 32 | 1 | 16 | 15 | 1 | 6,25 | 0,25 |
| 32 | 2 | 16 | 13 | 3 | 18,75 | 0,43 |
| 32 | 3 | 16 | 14 | 2 | 12,5 | 0,35 |
| 64 | 1 | 16 | 2 | 14 | 87,5 | 0,94 |
| 64 | 2 | 16 | 0 | 16 | 100 | 1,00 |
| 64 | 3 | 16 | 0 | 16 | 100 | 1,00 |
| 128 | 1 | 16 | 0 | 16 | 100 | 1,00 |
| 128 | 2 | 16 | 1 | 15 | 93,75 | 0,97 |
| 128 | 3 | 16 | 0 | 16 | 100 | 1,00 |
| 256 | 1 | 16 | 0 | 16 | 100 | 1,00 |
| 256 | 2 | 16 | 0 | 16 | 100 | 1,00 |
| 256 | 3 | 16 | 0 | 16 | 100 | 1,00 |

| **Bioassay *A. grandis* - Cry1Ia10 + Cry3Aa Proteins** | | | | | | |
| --- | --- | --- | --- | --- | --- | --- |
| Cry1Ia10 + Cry3Aa | | | | | | |
| treatment | repetition | total | alive | dead | Mort (%) | (X/100)^0.5 |
| Cont | 1 | 16 | 16 | 0 | 0 | 0,00 |
| Cont | 2 | 16 | 15 | 1 | 6,25 | 0,25 |
| Cont | 3 | 16 | 14 | 2 | 12,5 | 0,35 |
| 4 | 1 | 16 | 9 | 7 | 43,75 | 0,66 |
| 4 | 2 | 16 | 5 | 11 | 68,75 | 0,83 |
| 4 | 3 | 16 | 5 | 11 | 68,75 | 0,83 |
| 8 | 1 | 16 | 3 | 13 | 81,25 | 0,90 |
| 8 | 2 | 16 | 6 | 10 | 62,5 | 0,79 |
| 8 | 3 | 16 | 3 | 13 | 81,25 | 0,90 |
| 16 | 1 | 16 | 4 | 12 | 75 | 0,87 |
| 16 | 2 | 16 | 2 | 14 | 87,5 | 0,94 |
| 16 | 3 | 16 | 1 | 15 | 93,75 | 0,97 |
| 32 | 1 | 16 | 0 | 16 | 100 | 1,00 |
| 32 | 2 | 16 | 2 | 14 | 87,5 | 0,94 |
| 32 | 3 | 16 | 2 | 14 | 87,5 | 0,94 |
| 64 | 1 | 16 | 0 | 16 | 100 | 1,00 |
| 64 | 2 | 16 | 1 | 15 | 93,75 | 0,97 |
| 64 | 3 | 16 | 1 | 15 | 93,75 | 0,97 |
| 128 | 1 | 16 | 1 | 15 | 93,75 | 0,97 |
| 128 | 2 | 16 | 0 | 16 | 100 | 1,00 |
| 128 | 3 | 16 | 1 | 15 | 93,75 | 0,97 |
| 256 | 1 | 16 | 1 | 15 | 93,75 | 0,97 |
| 256 | 2 | 16 | 0 | 16 | 100 | 1,00 |
| 256 | 3 | 16 | 0 | 16 | 100 | 1,00 |

| **Bioassay *A. grandis* - Cry1Ia10 + Cry8B Proteins** | | | | | | |
| --- | --- | --- | --- | --- | --- | --- |
| Cry1Ia10 + Cry8B | | | | | | |
| treatment | repetition | total | alive | dead | Mort (%) | (X/100)^0.5 |
| Cont | 1 | 16 | 16 | 0 | 0 | 0,00 |
| Cont | 2 | 16 | 15 | 1 | 6,25 | 0,25 |
| Cont | 3 | 16 | 14 | 2 | 12,5 | 0,35 |
| 4 | 1 | 16 | 10 | 6 | 37,5 | 0,61 |
| 4 | 2 | 16 | 9 | 7 | 43,75 | 0,66 |
| 4 | 3 | 16 | 11 | 5 | 31,25 | 0,56 |
| 8 | 1 | 16 | 5 | 11 | 68,75 | 0,83 |
| 8 | 2 | 16 | 6 | 10 | 62,5 | 0,79 |
| 8 | 3 | 16 | 10 | 6 | 37,5 | 0,61 |
| 16 | 1 | 16 | 1 | 15 | 93,75 | 0,97 |
| 16 | 2 | 16 | 4 | 12 | 75 | 0,87 |
| 16 | 3 | 16 | 4 | 12 | 75 | 0,87 |
| 32 | 1 | 16 | 2 | 14 | 87,5 | 0,94 |
| 32 | 2 | 16 | 1 | 15 | 93,75 | 0,97 |
| 32 | 3 | 16 | 2 | 14 | 87,5 | 0,94 |
| 64 | 1 | 16 | 1 | 15 | 93,75 | 0,97 |
| 64 | 2 | 16 | 0 | 16 | 100 | 1,00 |
| 64 | 3 | 16 | 1 | 15 | 93,75 | 0,97 |
| 128 | 1 | 16 | 0 | 16 | 100 | 1,00 |
| 128 | 2 | 16 | 0 | 16 | 100 | 1,00 |
| 128 | 3 | 16 | 1 | 15 | 93,75 | 0,97 |
| 256 | 1 | 16 | 1 | 15 | 93,75 | 0,97 |
| 256 | 2 | 16 | 0 | 16 | 100 | 1,00 |
| 256 | 3 | 16 | 0 | 16 | 100 | 1,00 |

| **Bioassay *A. grandis* - Cry3Aa + Cry8B Proteins** | | | | | | |
| --- | --- | --- | --- | --- | --- | --- |
| Cry3Aa + Cry8B | | | | | | |
| treatment | repetition | total | alive | dead | Mort (%) | (X/100)^0.5 |
| Cont | 1 | 16 | 16 | 0 | 0 | 0,00 |
| Cont | 2 | 16 | 15 | 1 | 6,25 | 0,25 |
| Cont | 3 | 16 | 14 | 2 | 12,5 | 0,35 |
| 4 | 1 | 16 | 6 | 10 | 62,5 | 0,79 |
| 4 | 2 | 16 | 4 | 12 | 75 | 0,87 |
| 4 | 3 | 16 | 7 | 9 | 56,25 | 0,75 |
| 8 | 1 | 16 | 3 | 13 | 81,25 | 0,90 |
| 8 | 2 | 16 | 2 | 14 | 87,5 | 0,94 |
| 8 | 3 | 16 | 4 | 12 | 75 | 0,87 |
| 16 | 1 | 16 | 1 | 15 | 93,75 | 0,97 |
| 16 | 2 | 16 | 4 | 12 | 75 | 0,87 |
| 16 | 3 | 16 | 1 | 15 | 93,75 | 0,97 |
| 32 | 1 | 16 | 2 | 14 | 87,5 | 0,94 |
| 32 | 2 | 16 | 0 | 16 | 100 | 1,00 |
| 32 | 3 | 16 | 0 | 16 | 100 | 1,00 |
| 64 | 1 | 16 | 0 | 16 | 100 | 1,00 |
| 64 | 2 | 16 | 0 | 16 | 100 | 1,00 |
| 64 | 3 | 16 | 1 | 15 | 93,75 | 0,97 |
| 128 | 1 | 16 | 0 | 16 | 100 | 1,00 |
| 128 | 2 | 16 | 0 | 16 | 100 | 1,00 |
| 128 | 3 | 16 | 0 | 16 | 100 | 1,00 |
| 256 | 1 | 16 | 0 | 16 | 100 | 1,00 |
| 256 | 2 | 16 | 0 | 16 | 100 | 1,00 |
| 256 | 3 | 16 | 0 | 16 | 100 | 1,00 |

| **Bioassay *A. grandis* - IPD072Aa + Cry1Ia10 Proteins** | | | | | | |
| --- | --- | --- | --- | --- | --- | --- |
| IPD072Aa + Cry1Ia10 | | | | | | |
| treatment | repetition | total | alive | dead | Mort (%) | (X/100)^0.5 |
| Cont | 1 | 16 | 15 | 1 | 6,25 | 0,25 |
| Cont | 2 | 16 | 16 | 0 | 0 | 0,00 |
| Cont | 3 | 16 | 14 | 2 | 12,5 | 0,35 |
| 2 | 1 | 16 | 11 | 5 | 31,25 | 0,56 |
| 2 | 2 | 16 | 15 | 1 | 6,25 | 0,25 |
| 2 | 3 | 16 | 12 | 4 | 25 | 0,50 |
| 4 | 1 | 16 | 8 | 8 | 50 | 0,71 |
| 4 | 2 | 16 | 12 | 4 | 25 | 0,50 |
| 4 | 3 | 16 | 10 | 6 | 37,5 | 0,61 |
| 8 | 1 | 16 | 2 | 14 | 87,5 | 0,94 |
| 8 | 2 | 16 | 4 | 12 | 75 | 0,87 |
| 8 | 3 | 16 | 6 | 10 | 62,5 | 0,79 |
| 16 | 1 | 16 | 1 | 15 | 93,75 | 0,97 |
| 16 | 2 | 16 | 3 | 13 | 81,25 | 0,90 |
| 16 | 3 | 16 | 2 | 14 | 87,5 | 0,94 |
| 32 | 1 | 16 | 1 | 15 | 93,75 | 0,97 |
| 32 | 2 | 16 | 1 | 15 | 93,75 | 0,97 |
| 32 | 3 | 16 | 2 | 14 | 87,5 | 0,94 |

| **Bioassay *A. grandis* - IPD072Aa + Cry3Aa Proteins** | | | | | | |
| --- | --- | --- | --- | --- | --- | --- |
| IPD072Aa + Cry3Aa | | | | | | |
| treatment | repetition | total | alive | dead | Mort (%) | (X/100)^0.5 |
| Cont | 1 | 16 | 15 | 1 | 6,25 | 0,25 |
| Cont | 2 | 16 | 16 | 0 | 0 | 0,00 |
| Cont | 3 | 16 | 14 | 2 | 12,5 | 0,35 |
| 2 | 1 | 16 | 13 | 3 | 18,75 | 0,43 |
| 2 | 2 | 16 | 10 | 6 | 37,5 | 0,61 |
| 2 | 3 | 16 | 13 | 3 | 18,75 | 0,43 |
| 4 | 1 | 16 | 5 | 11 | 68,75 | 0,83 |
| 4 | 2 | 16 | 7 | 9 | 56,25 | 0,75 |
| 4 | 3 | 16 | 9 | 7 | 43,75 | 0,66 |
| 8 | 1 | 16 | 1 | 15 | 93,75 | 0,97 |
| 8 | 2 | 16 | 6 | 10 | 62,5 | 0,79 |
| 8 | 3 | 16 | 5 | 11 | 68,75 | 0,83 |
| 16 | 1 | 16 | 0 | 16 | 100 | 1,00 |
| 16 | 2 | 16 | 4 | 12 | 75 | 0,87 |
| 16 | 3 | 16 | 2 | 14 | 87,5 | 0,94 |
| 32 | 1 | 16 | 2 | 14 | 87,5 | 0,94 |
| 32 | 2 | 16 | 1 | 15 | 93,75 | 0,97 |
| 32 | 3 | 16 | 1 | 15 | 93,75 | 0,97 |

| **Bioassay *A. grandis* - IPD072Aa + Cry8B Proteins** | | | | | | |
| --- | --- | --- | --- | --- | --- | --- |
| IPD072Aa + Cry8B | | | | | | |
| treatment | repetition | total | alive | dead | Mort (%) | (X/100)^0.5 |
| Cont | 1 | 16 | 15 | 1 | 6,25 | 0,25 |
| Cont | 2 | 16 | 16 | 0 | 0 | 0,00 |
| Cont | 3 | 16 | 14 | 2 | 12,5 | 0,35 |
| 2 | 1 | 16 | 13 | 3 | 18,75 | 0,43 |
| 2 | 2 | 16 | 8 | 8 | 50 | 0,71 |
| 2 | 3 | 16 | 12 | 4 | 25 | 0,50 |
| 4 | 1 | 16 | 7 | 9 | 56,25 | 0,75 |
| 4 | 2 | 16 | 5 | 11 | 68,75 | 0,83 |
| 4 | 3 | 16 | 4 | 12 | 75 | 0,87 |
| 8 | 1 | 16 | 2 | 14 | 87,5 | 0,94 |
| 8 | 2 | 16 | 5 | 11 | 68,75 | 0,83 |
| 8 | 3 | 16 | 3 | 13 | 81,25 | 0,90 |
| 16 | 1 | 16 | 1 | 15 | 93,75 | 0,97 |
| 16 | 2 | 16 | 2 | 14 | 87,5 | 0,94 |
| 16 | 3 | 16 | 2 | 14 | 87,5 | 0,94 |
| 32 | 1 | 16 | 0 | 16 | 100 | 1,00 |
| 32 | 2 | 16 | 0 | 16 | 100 | 1,00 |
| 32 | 3 | 16 | 1 | 15 | 93,75 | 0,97 |

| **Bioassay *A. grandis* - PIP-47Aa + Cry1Ia10 Proteins** | | | | | | |
| --- | --- | --- | --- | --- | --- | --- |
| PIP-47Aa + Cry1Ia10 | | | | | | |
| treatment | repetition | total | alive | dead | Mort (%) | (X/100)^0.5 |
| Cont | 1 | 16 | 15 | 1 | 6,25 | 0,25 |
| Cont | 2 | 16 | 16 | 0 | 0 | 0,00 |
| Cont | 3 | 16 | 14 | 2 | 12,5 | 0,35 |
| 2 | 1 | 16 | 13 | 3 | 18,75 | 0,43 |
| 2 | 2 | 16 | 11 | 5 | 31,25 | 0,56 |
| 2 | 3 | 16 | 14 | 2 | 12,5 | 0,35 |
| 4 | 1 | 16 | 6 | 10 | 62,5 | 0,79 |
| 4 | 2 | 16 | 10 | 6 | 37,5 | 0,61 |
| 4 | 3 | 16 | 9 | 7 | 43,75 | 0,66 |
| 8 | 1 | 16 | 4 | 12 | 75 | 0,87 |
| 8 | 2 | 16 | 6 | 10 | 62,5 | 0,79 |
| 8 | 3 | 16 | 2 | 12 | 75 | 0,87 |
| 16 | 1 | 16 | 2 | 14 | 87,5 | 0,94 |
| 16 | 2 | 16 | 0 | 16 | 100 | 1,00 |
| 16 | 3 | 16 | 6 | 10 | 62,5 | 0,79 |
| 32 | 1 | 16 | 0 | 16 | 100 | 1,00 |
| 32 | 2 | 16 | 0 | 16 | 100 | 1,00 |
| 32 | 3 | 16 | 3 | 13 | 81,25 | 0,90 |

| **Bioassay *A. grandis* - PIP-47Aa + Cry3Aa Proteins** | | | | | | |
| --- | --- | --- | --- | --- | --- | --- |
| PIP-47Aa + Cry3Aa | | | | | | |
| treatment | repetition | total | alive | dead | Mort (%) | (X/100)^0.5 |
| Cont | 1 | 16 | 16 | 0 | 0 | 0,00 |
| Cont | 2 | 16 | 15 | 1 | 6,25 | 0,25 |
| Cont | 3 | 16 | 16 | 0 | 0 | 0,00 |
| 2 | 1 | 16 | 11 | 5 | 31,25 | 0,56 |
| 2 | 2 | 16 | 11 | 5 | 31,25 | 0,56 |
| 2 | 3 | 16 | 13 | 3 | 18,75 | 0,43 |
| 4 | 1 | 16 | 5 | 11 | 68,75 | 0,83 |
| 4 | 2 | 16 | 9 | 7 | 43,75 | 0,66 |
| 4 | 3 | 16 | 7 | 9 | 56,25 | 0,75 |
| 8 | 1 | 16 | 0 | 16 | 100 | 1,00 |
| 8 | 2 | 16 | 5 | 11 | 68,75 | 0,83 |
| 8 | 3 | 16 | 6 | 10 | 62,5 | 0,79 |
| 16 | 1 | 16 | 2 | 14 | 87,5 | 0,94 |
| 16 | 2 | 16 | 0 | 16 | 100 | 1,00 |
| 16 | 3 | 16 | 4 | 12 | 75 | 0,87 |
| 32 | 1 | 16 | 1 | 15 | 93,75 | 0,97 |
| 32 | 2 | 16 | 1 | 15 | 93,75 | 0,97 |
| 32 | 3 | 16 | 0 | 16 | 100 | 1,00 |

| **Bioassay *A. grandis* - PIP-47Aa + Cry8B Proteins** | | | | | | |
| --- | --- | --- | --- | --- | --- | --- |
| PIP-47Aa + Cry8B | | | | | | |
| treatment | repetition | total | alive | dead | Mort (%) | (X/100)^0.5 |
| Cont | 1 | 16 | 16 | 0 | 0 | 0,00 |
| Cont | 2 | 16 | 15 | 1 | 6,25 | 0,25 |
| Cont | 3 | 16 | 16 | 0 | 0 | 0,00 |
| 2 | 1 | 16 | 10 | 6 | 37,5 | 0,61 |
| 2 | 2 | 16 | 12 | 4 | 25 | 0,50 |
| 2 | 3 | 16 | 12 | 4 | 25 | 0,50 |
| 4 | 1 | 16 | 8 | 8 | 50 | 0,71 |
| 4 | 2 | 16 | 6 | 10 | 62,5 | 0,79 |
| 4 | 3 | 16 | 5 | 11 | 68,75 | 0,83 |
| 8 | 1 | 16 | 3 | 13 | 81,25 | 0,90 |
| 8 | 2 | 16 | 5 | 11 | 68,75 | 0,83 |
| 8 | 3 | 16 | 4 | 12 | 75 | 0,87 |
| 16 | 1 | 16 | 0 | 16 | 100 | 1,00 |
| 16 | 2 | 16 | 4 | 12 | 75 | 0,87 |
| 16 | 3 | 16 | 4 | 12 | 75 | 0,87 |
| 32 | 1 | 16 | 0 | 16 | 100 | 1,00 |
| 32 | 2 | 16 | 0 | 16 | 100 | 1,00 |
| 32 | 3 | 16 | 1 | 15 | 93,75 | 0,97 |

Table LC

| Cry1Ia10 |  |  |  | Cry3Aa |  |  |  | Cry8B |  |  |
| --- | --- | --- | --- | --- | --- | --- | --- | --- | --- | --- |
| ug/mL |  |  |  | ug/mL |  |  |  | ug/mL |  |  |
| Doses | Total | Dead |  | Doses | Total | Dead |  | Doses | Total | Dead |
| 0 | 48 | 1 |  | 0 | 48 | 1 |  | 0 | 48 | 1 |
| 4 | 48 | 10 |  | 4 | 48 | 12 |  | 4 | 48 | 17 |
| 8 | 48 | 18 |  | 8 | 48 | 27 |  | 8 | 48 | 29 |
| 16 | 48 | 36 |  | 16 | 48 | 36 |  | 16 | 48 | 37 |
| 32 | 48 | 42 |  | 32 | 48 | 42 |  | 32 | 48 | 42 |
| 64 | 48 | 44 |  | 64 | 48 | 44 |  | 64 | 48 | 46 |
| 128 | 48 | 46 |  | 128 | 48 | 47 |  | 128 | 48 | 47 |
| 256 | 48 | 47 |  | 256 | 48 | 47 |  | 256 | 48 | 48 |
|  |  |  |  |  |  |  |  |  |  |  |
|  |  |  |  |  |  |  |  |  |  |  |
| Cry1Ia10 + Cry3Aa | |  |  | Cry1Ia10 + Cry8B | |  |  | Cry3Aa + Cry8B | |  |
| ug/mL |  |  |  | ug/mL |  |  |  | ug/mL |  |  |
| Doses | Total | Dead |  | Doses | Total | Dead |  | Doses | Total | Dead |
| 0 | 48 | 3 |  | 0 | 48 | 3 |  | 0 | 48 | 3 |
| 4 | 48 | 29 |  | 4 | 48 | 18 |  | 4 | 48 | 31 |
| 8 | 48 | 36 |  | 8 | 48 | 27 |  | 8 | 48 | 39 |
| 16 | 48 | 41 |  | 16 | 48 | 39 |  | 16 | 48 | 42 |
| 32 | 48 | 44 |  | 32 | 48 | 43 |  | 32 | 48 | 46 |
| 64 | 48 | 46 |  | 64 | 48 | 46 |  | 64 | 48 | 47 |
| 128 | 48 | 46 |  | 128 | 48 | 47 |  | 128 | 48 | 48 |
| 256 | 48 | 47 |  | 256 | 48 | 47 |  | 256 | 48 | 48 |
|  |  |  |  |  |  |  |  |  |  |  |
|  |  |  |  |  |  | Table LC |  |  |  |  |
| IPD072Aa |  |  |  | PIP-47Aa |  |  |  | IPD072Aa + PIP-47Aa | | |
| ug/mL |  |  |  | ug/mL |  |  |  | ug/mL |  |  |
| Doses | Total | Dead |  | Doses | Total | Dead |  | Doses | Total | Dead |
| 0 | 48 | 2 |  | 0 | 48 | 2 |  | 0 | 48 | 2 |
| 4 | 48 | 6 |  | 4 | 48 | 8 |  | 4 | 48 | 16 |
| 8 | 48 | 15 |  | 8 | 48 | 13 |  | 8 | 48 | 25 |
| 16 | 48 | 30 |  | 16 | 48 | 25 |  | 16 | 48 | 39 |
| 32 | 48 | 41 |  | 32 | 48 | 30 |  | 32 | 48 | 45 |
| 64 | 48 | 42 |  | 64 | 48 | 43 |  | 64 | 48 | 46 |
| 128 | 48 | 46 |  | 128 | 48 | 46 |  | 128 | 48 | 47 |
| 256 | 48 | 46 |  | 256 | 48 | 47 |  | 256 | 48 | 47 |
|  |  |  |  |  |  |  |  |  |  |  |
|  |  |  |  |  |  |  |  |  |  |  |
| IPD072Aa + Cry1Ia10 | |  |  | IPD072Aa + Cry3Aa | |  |  | IPD072Aa + Cry8B | |  |
| ug/mL |  |  |  | ug/mL |  |  |  | ug/mL |  |  |
| Doses | Total | Dead |  | Doses | Total | Dead |  | Doses | Total | Dead |
| 0 | 48 | 3 |  | 0 | 48 | 3 |  | 0 | 48 | 3 |
| 2 | 48 | 10 |  | 2 | 48 | 12 |  | 2 | 48 | 15 |
| 4 | 48 | 18 |  | 4 | 48 | 27 |  | 4 | 48 | 32 |
| 8 | 48 | 36 |  | 8 | 48 | 36 |  | 8 | 48 | 38 |
| 16 | 48 | 42 |  | 16 | 48 | 42 |  | 16 | 48 | 43 |
| 32 | 48 | 44 |  | 32 | 48 | 44 |  | 32 | 48 | 47 |
|  |  |  |  |  |  |  |  |  |  |  |
|  |  |  |  |  |  | Table LC |  |  |  |  |
| PIP-47Aa + Cry1Ia10 | |  |  | PIP-47Aa + Cry3Aa | |  |  | PIP-47Aa + Cry8B | |  |
| ug/mL |  |  |  | ug/mL |  |  |  | ug/mL |  |  |
| Doses | Total | Dead |  | Doses | Total | Dead |  | Doses | Total | Dead |
| 0 | 48 | 3 |  | 0 | 48 | 1 |  | 0 | 48 | 1 |
| 2 | 48 | 10 |  | 2 | 48 | 13 |  | 2 | 48 | 14 |
| 4 | 48 | 23 |  | 4 | 48 | 28 |  | 4 | 48 | 29 |
| 8 | 48 | 34 |  | 8 | 48 | 37 |  | 8 | 48 | 36 |
| 16 | 48 | 40 |  | 16 | 48 | 42 |  | 16 | 48 | 40 |
| 32 | 48 | 45 |  | 32 | 48 | 46 |  | 32 | 48 | 47 |

| **Mortality of *Anthonomus grandis* neonate larvae** | | | | | | | |
| --- | --- | --- | --- | --- | --- | --- | --- |
| treatment | concentration | repetition | total | alive | dead | Mort (%) | (X/100)^0.5 |
| IPD072Aa | 14,24 | 1 | 16 | 42 | 6 | 37,5 | 0,61237244 |
| IPD072Aa | 14,24 | 2 | 16 | 39 | 9 | 56,25 | 0,75 |
| IPD072Aa | 14,24 | 3 | 16 | 38 | 10 | 62,5 | 0,79056942 |
| PIP-47Aa | 17,71 | 1 | 16 | 44 | 4 | 25 | 0,5 |
| PIP-47Aa | 17,71 | 2 | 16 | 37 | 11 | 68,75 | 0,8291562 |
| PIP-47Aa | 17,71 | 3 | 16 | 41 | 8 | 50 | 0,70710678 |
| Cry1Ia10 | 10,16 | 1 | 16 | 41 | 7 | 43,75 | 0,66143783 |
| Cry1Ia10 | 10,16 | 2 | 16 | 41 | 7 | 43,75 | 0,66143783 |
| Cry1Ia10 | 10,16 | 3 | 16 | 40 | 8 | 50 | 0,70710678 |
| Cry3Aa | 7,82 | 1 | 16 | 36 | 12 | 75 | 0,8660254 |
| Cry3Aa | 7,82 | 2 | 16 | 42 | 6 | 37,5 | 0,61237244 |
| Cry3Aa | 7,82 | 3 | 16 | 42 | 6 | 37,5 | 0,61237244 |
| Cry8B | 6,35 | 1 | 16 | 39 | 9 | 56,25 | 0,75 |
| Cry8B | 6,35 | 2 | 16 | 37 | 11 | 68,75 | 0,8291562 |
| Cry8B | 6,35 | 3 | 16 | 42 | 6 | 37,5 | 0,61237244 |
| IPD072Aa/PIP-47Aa | 14,24 + 17,71 | 1 | 16 | 33 | 15 | 93,75 | 0,96824584 |
| IPD072Aa/PIP-47Aa | 14,24 + 17,71 | 2 | 16 | 37 | 11 | 68,75 | 0,8291562 |
| IPD072Aa/PIP-47Aa | 14,24 + 17,71 | 3 | 16 | 36 | 12 | 75 | 0,8660254 |
| Cry1Ia10/Cry3Aa | 10,76 + 7,82 | 1 | 16 | 32 | 16 | 100 | 1 |
| Cry1Ia10/Cry3Aa | 10,76 + 7,82 | 2 | 16 | 39 | 9 | 56,25 | 0,75 |
| Cry1Ia10/Cry3Aa | 10,76 + 7,82 | 3 | 16 | 34 | 14 | 87,5 | 0,93541435 |
| Cry1Ia10/Cry8B | 10,76 + 6,35 | 1 | 16 | 41 | 7 | 43,75 | 0,66143783 |
| Cry1Ia10/Cry8B | 10,76 + 6,35 | 2 | 16 | 39 | 9 | 56,25 | 0,75 |
| Cry1Ia10/Cry8B | 10,76 + 6,35 | 3 | 16 | 45 | 3 | 18,75 | 0,4330127 |
| Cry3Aa/Cry8B | 7,82 + 6,35 | 1 | 16 | 33 | 15 | 93,75 | 0,96824584 |
| Cry3Aa/Cry8B | 7,82 + 6,35 | 2 | 16 | 34 | 14 | 87,5 | 0,93541435 |
| Cry3Aa/Cry8B | 7,82 + 6,35 | 3 | 16 | 38 | 10 | 62,5 | 0,79056942 |
| IPD072Aa/Cry1Ia10 | 14,24 + 10,16 | 1 | 16 | 35 | 13 | 81,25 | 0,90138782 |
| IPD072Aa/Cry1Ia10 | 14,24 + 10,16 | 2 | 16 | 36 | 12 | 75 | 0,8660254 |
| IPD072Aa/Cry1Ia10 | 14,24 + 10,16 | 3 | 16 | 35 | 13 | 81,25 | 0,90138782 |
| IPD072Aa/Cry3Aa | 14,24 + 7,82 | 1 | 16 | 33 | 15 | 93,75 | 0,96824584 |
| IPD072Aa/Cry3Aa | 14,24 + 7,82 | 2 | 16 | 37 | 11 | 68,75 | 0,8291562 |
| IPD072Aa/Cry3Aa | 14,24 + 7,82 | 3 | 16 | 36 | 12 | 75 | 0,8660254 |
| IPD072Aa/Cry8B | 14,24 + 6,35 | 1 | 16 | 39 | 9 | 56,25 | 0,75 |
| IPD072Aa/Cry8B | 14,24 + 6,35 | 2 | 16 | 36 | 12 | 75 | 0,8660254 |
| IPD072Aa/Cry8B | 14,24 + 6,35 | 3 | 16 | 32 | 16 | 100 | 1 |
| PIP-47Aa/Cry1Ia10 | 17,71 + 10,16 | 1 | 16 | 31 | 17 | 106,25 | 1,03077641 |
| PIP-47Aa/Cry1Ia10 | 17,71 + 10,16 | 2 | 16 | 39 | 9 | 56,25 | 0,75 |
| PIP-47Aa/Cry1Ia10 | 17,71 + 10,16 | 3 | 16 | 38 | 10 | 62,5 | 0,79056942 |
| PIP-47Aa/Cry3Aa | 17,71 + 7,82 | 1 | 16 | 36 | 12 | 75 | 0,8660254 |
| PIP-47Aa/Cry3Aa | 17,71 + 7,82 | 2 | 16 | 33 | 15 | 93,75 | 0,96824584 |
| PIP-47Aa/Cry3Aa | 17,71 + 7,82 | 3 | 16 | 38 | 10 | 62,5 | 0,79056942 |
| PIP-47Aa/Cry8B | 17,71 + 6,35 | 1 | 16 | 37 | 11 | 68,75 | 0,8291562 |
| PIP-47Aa/Cry8B | 17,71 + 6,35 | 2 | 16 | 37 | 11 | 68,75 | 0,8291562 |
| PIP-47Aa/Cry8B | 17,71 + 6,35 | 3 | 16 | 33 | 15 | 93,75 | 0,96824584 |
