# Supplementary material for: Interaction of insecticidal proteins from Pseudomonas spp. and Bacillus thuringiensis for boll weevil management
Source: PLoS One. 2023 Nov 30;18(11):e0294654. doi: 10.1371/journal.pone.0294654 (PMC10688866; doi:10.1371/journal.pone.0294654)

**Figure 1 gel
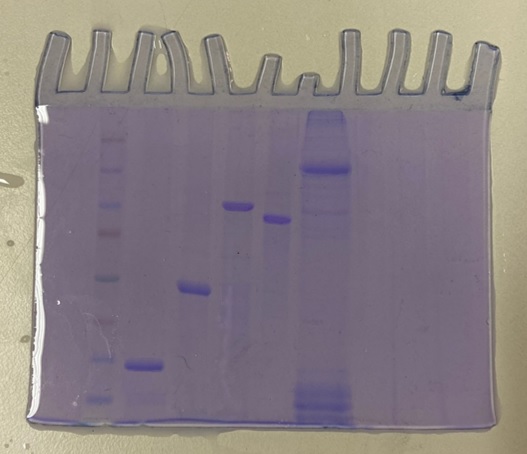
**

**Figure 1 gel**


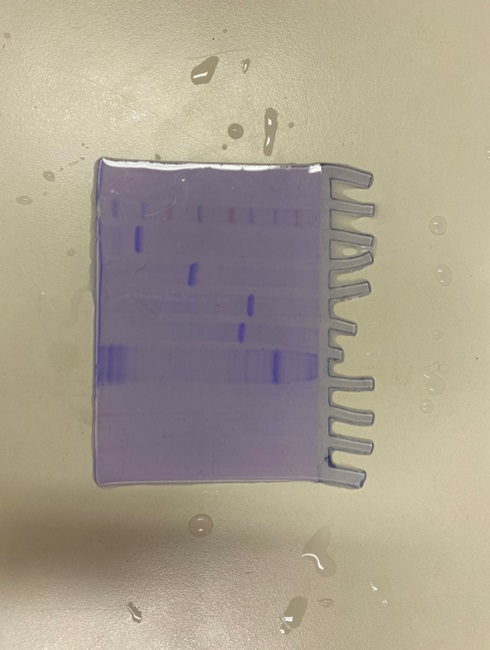


**Figure 1 gel**


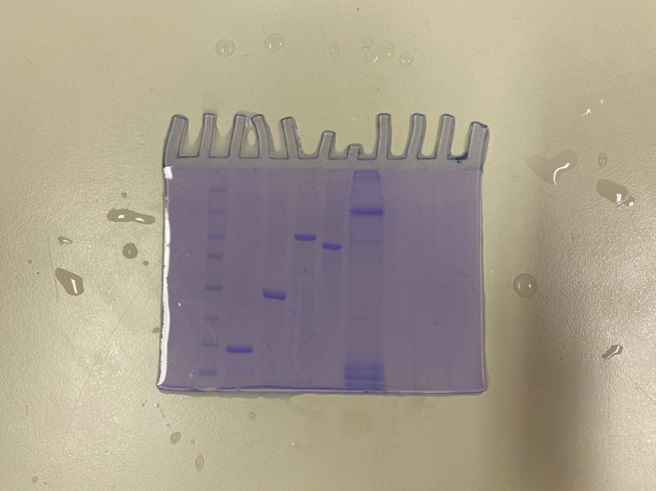


**Molecular marker “Spectra^TM^ Multicolor Broad Range Protein Ladder”**
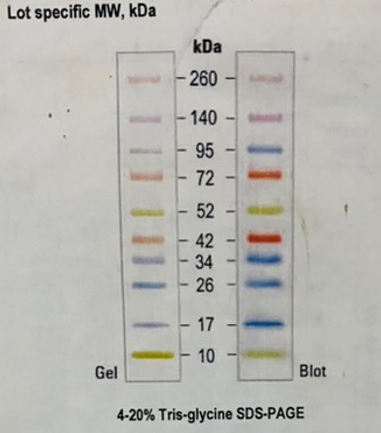

Supplement: S1 Raw images — (DOCX) [file pone.0294654.s002.docx]
