# Supplementary material for: Interaction of insecticidal proteins from Pseudomonas spp. and Bacillus thuringiensis for boll weevil management
Source: PLoS One. 2023 Nov 30;18(11):e0294654. doi: 10.1371/journal.pone.0294654 (PMC10688866; doi:10.1371/journal.pone.0294654)
Supplement: Attachment — Submitted filename: Response to Reviewers.docx [file pone.0294654.s003.docx]

Jaboticabal – Brazil, August 31, 2023.

Dr. Asaduzzaman Shishir

Academic Editor of Revista PLOS ONE

Dear Dr. Shishir,

The academic editor and reviewers made great recommendations and suggestions in the first version of the manuscript, which motivated us to improve the paper. The academic editor and reviewers appointed some points, which were mostly adopted in the new version. The suggestion that we that we do not agree with was justified in our replies.

Please, find below our detailed replies to the comments and suggestions of the academic editor and reviewers. In advance, we thank you and the reviewers for your availability to review our paper.

**Replies for the academic editor**

**Academic editor:** Please ensure that your manuscript meets PLOS ONE's style requirements, including those for file naming.

**Authors:** We meet the requirements and style of PLOS ONE for article production.

**Academic editor:** We note that the grant information you provided in the ‘Funding Information’ and ‘Financial Disclosure’ sections do not match.

**Authors:** We have corrected the grant information provided in the 'Funding Information' and 'Financial Disclosure' sections.

**Academic editor:** In your Data Availability statement, you have not specified where the minimal data set underlying the results described in your manuscript can be found.

**Authors:** We uploaded the minimum set of data from our study as supporting information, including the underlying data used to reach the conclusions drawn in the manuscript.

**Academic editor:** PLOS ONE now requires that authors provide the original uncropped and unadjusted images underlying all blot or gel results reported in a submission’s figures or Supporting Information files.

**Authors:** We provide the original underlying images for all gel data reported in our manuscript as supporting information.

**Academic editor:** Please remove your figures from within your manuscript file, leaving only the individual TIFF/EPS image files, uploaded separately. These will be automatically included in the reviewers’ PDF.

**Authors:** We removed the image from our manuscript.

**Replies for the first reviewer**

**Reviewer:** A conclusion with future direction(s) must be included in the manuscript before publication

**Authors:** We included a conclusion with future direction in our manuscript.

**Reviewer:** There is few minor gramtical mistakes. Those also should be addressed.

**Authors:** We conducted a review throughout the manuscript text to eliminate grammatical errors.

**Replies for the second reviewer**

**Reviewer:** In the abstract and introduction, the authors have raised several confusions regarding the boll weevil. Firstly, *A. grandis* is the most important cotton pest in the Americas, not worldwide. Additionally, there are no commercially available transgenic crops or pesticides based on Bt Cry toxins specifically targeting the boll weevil, so the issue of its resistance to Bt toxins does not exist. These statements need to be corrected.

**Authors:** We corrected the information in the abstract and introduction of our article, emphasizing that *A. grandis* is a significant pest in the Americas. We also rectified the information regarding resistance to Bt toxins.

**Reviewer:** The introduction is overly focused on Brazil, while it should be noted that A. grandis is an important pest from the US to Argentina.

**Authors:** In the introduction, we emphasized that *A. grandis* is a significant pest for Brazil and in countries across the Americas.

**Reviewer:** In lines 79-80, the authors state that "Upon ingestion of these proteins, the high intestinal pH of susceptible larvae activates a protoxin that binds to specific receptors…". This statement is true only for lepidopteran insects; for other insects, such as coleopterans, the mechanism is slightly different. This distinction should be clarified in the text.

**Authors:** We clarified the distinction between the uptake of Bt proteins by insects of the Lepidopteran and Coleopteran orders.

**Reviewer:** The statistical analysis of Table 1 data is misinterpreted. The authors claim certain statistical differences that are not valid. For example, at LC_50_, Cry1Ia10 overlaps with all the other proteins, and at LC_90_, only Cry8 and PIP-47 show significant differences. These errors should be corrected, along with the sentences referring to these results throughout the manuscript.

**Authors:** We corrected the statistical errors presented in Table 1, as well as the sentences referring to these results throughout the manuscript.

**Reviewer:** The discussion section needs to be reformulated as it contains excessive repetition.

**Authors:** We restructured the discussion of our article.

**Reviewer:** Additionally, I believe the text would benefit from an additional round of language corrections.

**Authors:** We conducted a review throughout the manuscript text to eliminate grammatical errors.

**Minor Issues**

Line 34: Change "Coleoptera" to "coleopteran."

**Authors:** We made this change.

Line 37: Specify which Cry8 variant was used: Cry8Aa, B, or C?

**Authors:** We specified which variant of the Cry8 protein we used.

Line 83: Change "action" to "activity."

**Authors:** We made this change.

Line 101: Change "methods" to "agents."

**Authors:** We made this change.

Line 195: Change "Assay of interactions between proteins against *Anthonomus grandis*" to "Assay of different protein combinations against *Anthonomus grandis*."

**Authors:** We made this change.

Line 268: In Table 1, change "IC" to "CI."

**Authors:** We made this change.

Lines 310 and 314: Change "FS" to "SF."

**Authors:** We made this change.

Lines 327-330: This sentence should be moved to the beginning of the paragraph.

**Authors:** We made this change.

Lines 343-344: Were all these toxins expressed, purified, and tested against coleopteran insects? Or were strains that harbor these protein genes found to be toxic to coleopterans?

**Authors:** We made this change, indicating that it is the strains harboring these protein genes that were considered toxic to the coleopterans.

Line 345: Change "Coleoptera" to "coleopteran."

**Authors:** We made this change.

Lines 353-354: Correct the decimal numbers; some use periods, while others use commas as separators.

**Authors:** We made this change.

Lines 371-375: Please verify if the bioassay conditions were the same for the compared experiments.

**Authors:** We reviewed the conditions of the compared bioassays and made modifications to the text.

Lines 377-378: I fail to see how this sentence contributes to the discussion. The cited reference pertains to a different toxin expressed in a different system, making it inappropriate for dose comparison.

**Authors:** We removed this sentence as it was inappropriate for the intended comparison.

Lines 386-387: This sentence lacks meaning.

**Authors:** We modified the paragraph to make more sense.

Lines 396-399: Were the tested larvae in the same instar or size in these experiments? Larval size can influence LC values, so it is important to consider this when making comparisons.

**Authors:** The tested larvae were in the same instar, this information has been added to the text.

Line 425: Cry1Ia is highly toxic to *A. grandis* compared to which other Cry protein? For comparison, in Lepidoptera, the LC_50_ values are typically in ng, indicating a much lower dosage is required to kill the insect larvae.

**Authors:** The text has been corrected, indicating that the Cry1I protein is toxic to *A. grandis*.

Line 431: The authors could further explore the competition of toxins for binding sites in the discussion.

**Authors:** As we still don't know whether our tested proteins against *A. grandis* actually compete for the same binding sites, we didn't delve into this exploration in the discussion. However, we have left, for future perspectives, the suggestion that research should be conducted through binding assays to elucidate the mode of action of these toxins, in order to identify specific membrane receptors and the possibility of competition for the same binding site among the proteins.

Line 442: Put "Lymantria dispar" in italics.

**Authors:** We made this change.

Line 460: What is "Howardi" referring to?

**Authors:** It should be *Diabrotica undecimpunctata howardi*, we made this correction in the text.

In the citations and references, please avoid the use of Master's and PhD theses. Instead, search for the related published articles that have undergone multiple rounds of peer review.

**Authors:** We made this recommendation.
