# Supplementary material for: Interaction of insecticidal proteins from Pseudomonas spp. and Bacillus thuringiensis for boll weevil management
Source: PLoS One. 2023 Nov 30;18(11):e0294654. doi: 10.1371/journal.pone.0294654 (PMC10688866; doi:10.1371/journal.pone.0294654)
Supplement: Attachment — Submitted filename: Response to Reviewers.docx [file pone.0294654.s004.docx]

Jaboticabal – Brazil, November 03, 2023.

Dr. Asaduzzaman Shishir

Academic Editor of Revista PLOS ONE

Dear Dr. Shishir,

The academic editor and the reviewers made great new recommendations and suggestions in this new version of the manuscript, which motivated us to improve the paper. The academic editor and the reviewers appointed some points, which were adopted in the new version.

Please, find below our detailed replies to the comments and suggestions of the academic editor and reviewers. In advance, we would like to thank you and the reviewers for your availability to review our paper.

**Replies for the academic editor**

**Academic editor:** Please review your reference list to ensure that it is complete and correct.

**Authors:** We have reviewed the entire list of references and added new references to the article, and some were corrected, namely:

References that were included:

ICAC - International Cotton Advisory Committee. Cotton This Month. Available at: https:// https://www.icac.org/Content/PublicationsPdf%20Files/c2e2b3f4_f2c3_48b7_834b_eb5e3e071695/CTM_2022_02_01.pdf.pdf. Accessed on: sept 24, 2023.

ABRAPA - Brazilian Association of Cotton Producers. Management Report 2021-2022. 207p. 2022. Available at: https://abapa.com.br/wp-content/uploads/2023/04/relatorio-de-gestao-2021-2022.pdf. Accessed on: sept 24, 2023.

EMBRAPA - Brazilian Agricultural Research Corporation. Available at: https://www.cnpa.embrapa.br/noticias/2013/noticia_20130807.html. Accessed on: sept 26, 2023.

Astriani M, Zubaidah S, Abadi AL, Suarsini, E. *Pseudomonas plecoglossicida* as a novel bacterium for phosphate solubilizing and indole-3-acetic acid-producing from soybean rhizospheric soils of East Java, Indonesia. **Biodiversitas Journal of Biological Diversity**. 2020; v. 21, n. 2, p. 578-586. doi: 10.13057/biodiv/d210220

Verma P, Yadav AN, Kumar V, Singh D P, Saxena, AK. Beneficial plant-microbes interactions: biodiversity of microbes from diverse extreme environments and its impact for crop improvement. **Plant-Microbe Interactions in Agro-Ecological Perspectives: volume 2: Microbial Interactions and Agro-Ecological Impacts**, 2017; p. 543-580.

Zhang Y, Zheng G, Tan J, Li C, Cheng L. Cloning and characterization of a novel cry8Ab1 gene from *Bacillus thuringiensis* strain B-JJX with specific toxicity to scarabaeid (Coleoptera: Scarabaeidae) larvae. **Microbiological research**. 2013; v. 168, n. 8, p. 512-517. doi: https://doi.org/10.1016/j.micres.2013.03.003

Gassmann AJ. Field-evolved resistance to Bt maize by western corn rootworm: Predictions from the laboratory and effects in the field. **Journal of Invertebrate Pathology**. 2012; v. 110, n. 3, p. 287-293. doi: 10.1016/j.jip.2012.04.006

Crickmore N, Berry C, Panneerselvam S, Mishra R, Connor TR, Bonning BC. A structure-based nomenclature for Bacillus thuringiensis and other bacteria-derived pesticidal proteins. Journal of Invertebrate Pathology. 2020; v. 186:107438. doi: 10.1016/j.jip.2020.107438

References that were corrected:

USDA - United States Department of Agriculture. Foreign agricultural service. Cotton: World Markets and Trade. Available at: https://apps.fas.usda.gov/psdonline/circulars/cotton.pdf. Accessed on: sept 23, 2023.

MEYER, L.A. Cotton and wool outlook. CWS-21g. Washington: USDA-ERS, July 2021. 8p. Available at: https://www.ers.usda.gov/webdocs/outlooks/101610/cws-21g.pdf?v=9514. Accessed on: sept 23, 2023.

We inserted the DOI in each reference, when available.

**Replies for the second reviewer**

**Reviewer:** All of the aforementioned concerns were effectively resolved. Only one last thing, the letter denoting statistical difference for Cry3Aa in table 1 should be revised from 'a' to 'ab'.

**Authors:** We corrected the statistical errors presented in Table 1, as well as the sentences referring to these results throughout the manuscript.

**NOTE:** While revising your submission, please upload your figure files to the Preflight Analysis and Conversion Engine (PACE) digital diagnostic tool, <https://pacev2.apexcovantage.com/>. PACE helps ensure that figures meet PLOS requirements

**Authors:** We loaded the figure file into the Preflight Analysis and Conversion Engine (PACE) digital diagnostic tool.
